# Analysis of Dissolution, Pharmacokinetic, and Bone-Protective Differences Among Calcium Formulations with Different Dosage Forms and Calcium Sources

**DOI:** 10.3390/ph19081172

**Published:** 2026-07-27

**Authors:** Mengxi Wang, Shuo Liu, Guang Wei, Haiyang Wang, Zewei Huang, Yang Ding, Guochen Han

**Affiliations:** 1 State Key Laboratory of Natural Medicines, Department of Pharmaceutics, China Pharmaceutical University, Nanjing 210009, China; wmx202108@163.com (M.W.); ls2608732869@163.com (S.L.); 15951073609@126.com (G.W.); 3325010507@stu.cpu.edu.cn (H.W.); 2 Sichuan Institute of Food Inspection, Chengdu 610097, China; huangzewei@scsspjyyjy.wecom.work

**Keywords:** calcium formulations, dosage forms, dissolution, pharmacokinetics, pharmacodynamics, osteoporosis

## Abstract

**Background**: Calcium formulations are important nutritional interventions for improving insufficient calcium intake and assisting in the prevention and treatment of osteoporosis. In vitro and in vivo performance may be influenced by dosage form, calcium source, and formulation composition. In this study, an integrated approach combining in vitro dissolution, pharmacokinetic, and pharmacodynamic evaluations was used to compare the comprehensive performance of nine calcium formulations and related functional components. **Methods**: The nine samples were coded as CS-1 to CS-9 according to a predefined order. Calcium release characteristics were determined under simulated gastrointestinal pH conditions using a flow-through cell system. Calcium concentrations in rat plasma, feces, and urine were measured by inductively coupled plasma optical emission spectrometry (ICP-OES) to evaluate calcium exposure, excretion, and retention. Meanwhile, the bone-protective effects of different calcium preparations were assessed in a retinoic acid-induced osteoporotic ICR mouse model, and the pharmacodynamic performance of calcium citrate and various bone-derived peptide preparations was investigated in a calcium-deficient female Sprague–Dawley (SD) rat model. **Results**: CS-1 exhibited rapid and nearly complete calcium release in simulated gastric fluid and showed a relatively favorable dissolution profile under sequential gastrointestinal pH conditions. Calcium balance analysis indicated that calcium retention in rats was relatively higher after administration of CS-1. Animal experiments showed that different calcium sources and bone-derived peptide preparations improved bone-related parameters to varying degrees, among which CS-1 produced relatively consistent improvements in bone microarchitecture and biomechanical properties. As a functional component of the CS-1 formulation, salmon bone-derived ingredients showed activity in the comparative pharmacodynamic evaluation, suggesting that they may contribute to the overall bone-protective effect. **Conclusions**: Different calcium formulations showed distinct profiles in dissolution behavior, calcium retention, and bone-protective effects. Among the tested samples, CS-1 demonstrated relatively favorable overall performance, which may be related to its liquid dosage form and salmon bone-derived functional components.

## 1. Introduction

Currently, commercially available calcium formulations are diverse and include various dosage forms, such as tablets, capsules, powders, chewable tablets, and liquid preparations [1]. Common calcium sources include calcium carbonate, calcium citrate, and other composite calcium sources [2]. Different products vary in calcium source, dosage form, excipient composition, disintegration behavior, and dissolution characteristics. These formulation-related factors may affect the release of soluble calcium in the gastrointestinal tract, while the formation of soluble calcium is an important prerequisite for intestinal absorption [3]. Calcium absorption is jointly regulated by active transport and passive diffusion and is influenced by multiple factors, including vitamin D status, gastric acidity, dietary components, and endogenous calcium homeostasis. Therefore, the declared calcium content of a formulation cannot fully reflect the actual in vivo performance of calcium formulations, and their comparative evaluation should integrate analyses of in vitro dissolution behavior as well as in vivo calcium exposure, excretion, and retention characteristics [4,5].

In addition to conventional calcium salts, functional ingredients containing bioactive peptides have also attracted attention in the field of bone health [6]. Collagen peptides and bone-derived peptides may affect bone metabolism by providing amino acid substrates required for collagen synthesis, modulating the activities of osteoblasts and osteoclasts, or promoting calcium utilization [7,8,9]. Some clinical and animal studies have suggested that collagen peptides, particularly when combined with calcium or other bone-related nutrients, may have beneficial effects on bone mineral density or bone turnover-related indicators [10,11,12]. However, in composite calcium formulations, the contribution of peptide-based functional ingredients to the overall bone-protective effect remains insufficiently clarified, and the relationship between bone peptides from different sources and the characteristics of calcium preparations requires further investigation [6,12].

The in vivo evaluation of calcium preparations has certain distinctive challenges because calcium itself is an endogenous ion under strict physiological regulation [13,14]. Plasma calcium concentrations are controlled by multiple mechanisms, including intestinal absorption, renal excretion, skeletal storage, and hormonal regulation, and are usually maintained within a relatively narrow range [13,14]. Therefore, reliance solely on conventional pharmacokinetic parameters may not fully characterize the in vivo behavior of different calcium formulations [13,15,16]. Combining analysis of plasma calcium exposure with fecal and urinary calcium excretion can provide a more comprehensive evaluation of calcium absorption, excretion, and retention. At the same time, pharmacodynamic endpoints such as bone mineral deposition, trabecular microarchitecture, and biomechanical properties are needed to further determine whether differences in dissolution behavior and calcium retention capacity can be translated into actual bone-protective effects [17,18]. The retinoic acid-induced osteoporotic animal model can lead to deterioration of bone microarchitecture and reduction in bone mechanical strength [19]. It has been used to evaluate bone loss and the bone-protective effects of nutritional or pharmacological interventions, and is suitable for comparative studies of candidate bone health products [19].

Based on this background, this study employed a purposive sampling strategy and selected nine commercially available calcium formulations with representative differences for comparison. All nine samples selected for this study were commercially available products with clearly labeled ingredients and dosage forms. Sample selection was primarily based on representativeness and formulation differences, covering liquid formulations, tablets, and soft capsules, as well as calcium citrate, milk calcium, composite calcium, and compound formulations fortified with vitamin D and vitamin K/K_2_. Among these, CS-1 combines salmon bone-derived ingredients with a liquid formulation, serving as a comparison group distinct from traditional tablets and soft capsules. Accordingly, we hypothesized that differences in dosage form, calcium source, and functional ingredients would be associated with distinct profiles of calcium release, apparent calcium utilization, and bone-protective efficacy.

In the present study, a flow-through cell system was employed to evaluate the calcium release profiles of the tested formulations under simulated gastrointestinal conditions [20,21,22]. Inductively coupled plasma optical emission spectrometry (ICP-OES) was further applied to quantify calcium levels in plasma, feces, and urine, thereby characterizing systemic calcium exposure, excretion, and retention in vivo [23,24]. Subsequently, the pharmacodynamic effects of different calcium formulations were investigated in an osteoporosis animal model, with particular emphasis on bone microarchitecture and biomechanical properties. In parallel, a calcium-deficient animal model was used to compare the pharmacological effects of calcium citrate and various bone-derived peptide formulations. Previous studies have indicated that fish and bone-derived peptides or peptide–calcium complexes may enhance calcium solubility or absorption and improve bone-related outcomes in calcium-deficient or ovariectomized rodents [25]. Collectively, this study provides a comprehensive evaluation of different calcium formulations from three complementary perspectives—in vitro calcium release behavior, in vivo calcium disposition and apparent retention, and pharmacodynamic efficacy—and may provide experimental evidence for the optimization of calcium formulation design and functional evaluation.

## 2. Results

### 2.1. In Vitro Dissolution Profiles of Calcium Formulations Under Simulated Gastrointestinal Conditions

#### 2.1.1. Calcium Release Under a Single Simulated Gastric pH Condition

**Figure 1:**
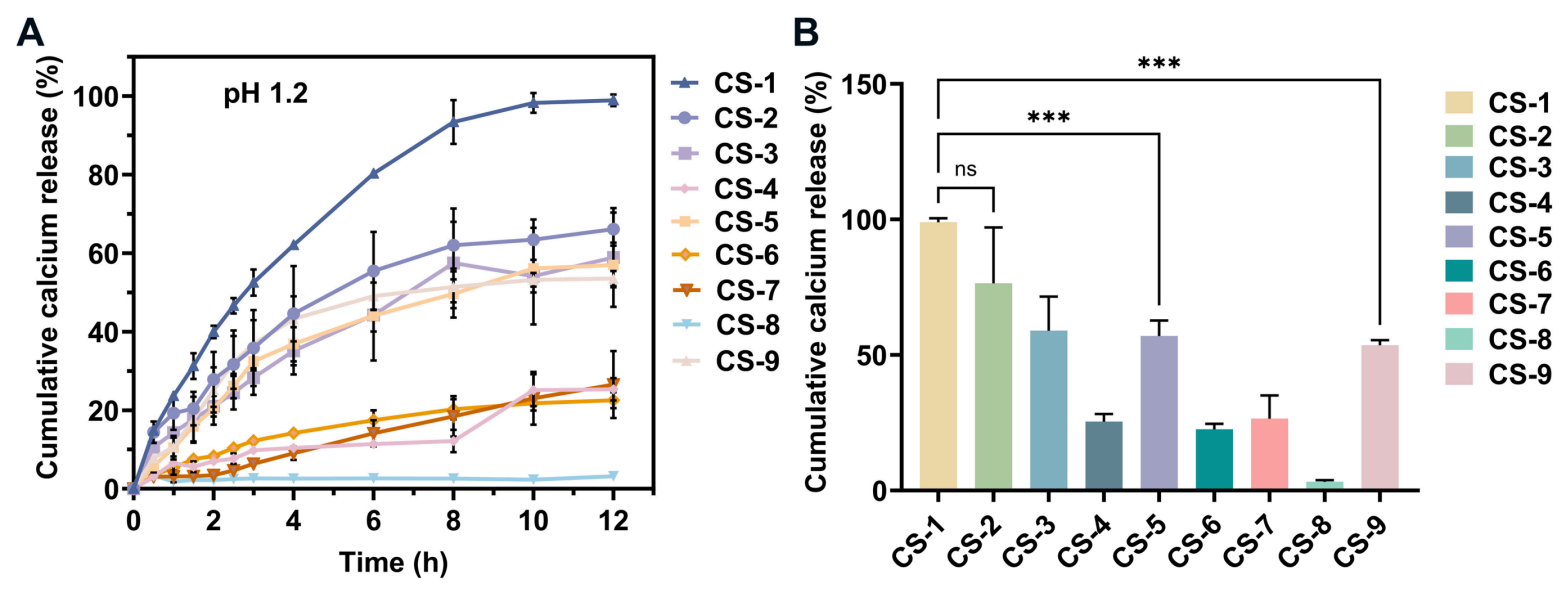
Cumulative calcium release profiles of different calcium formulations under simulated gastric conditions at pH 1.2. (**A**) Time-dependent cumulative calcium release profiles. (**B**) Cumulative calcium release for 12 h. Data are presented as mean ± SD (*n* = 3) *** *p* < 0.001; ns, not significant.

The dissolution behavior of the nine calcium formulations was first evaluated in pH 1.2 hydrochloric acid medium to simulate the acidic gastric environment. As shown in Figure 1A, the tested samples exhibited markedly different calcium release profiles under this condition. Among them, CS-1 displayed the fastest and most complete release, with cumulative calcium release increasing sharply during the early stage and approaching a plateau at nearly complete release within 6–8 h. This trend indicates that most of the available calcium in CS-1 was rapidly released under acidic conditions.

As further illustrated in Figure 1A, CS-2, CS-3, CS-4, and CS-9 showed progressive calcium release over time, but their final cumulative release values remained lower than that of CS-1. In contrast, CS-5, CS-6, CS-7, and CS-8 exhibited slower and less complete dissolution, with limited calcium release even after 12 h (Figure 1B). These differences suggest that dosage form and formulation characteristics substantially influenced calcium dissolution in the simulated gastric environment, with samples showing rapid dispersion or dissolution generally achieving higher calcium release than those with slower-disintegrating solid forms.

#### 2.1.2. Calcium Release Under Sequential Gastrointestinal pH Conditions

**Figure 2:**
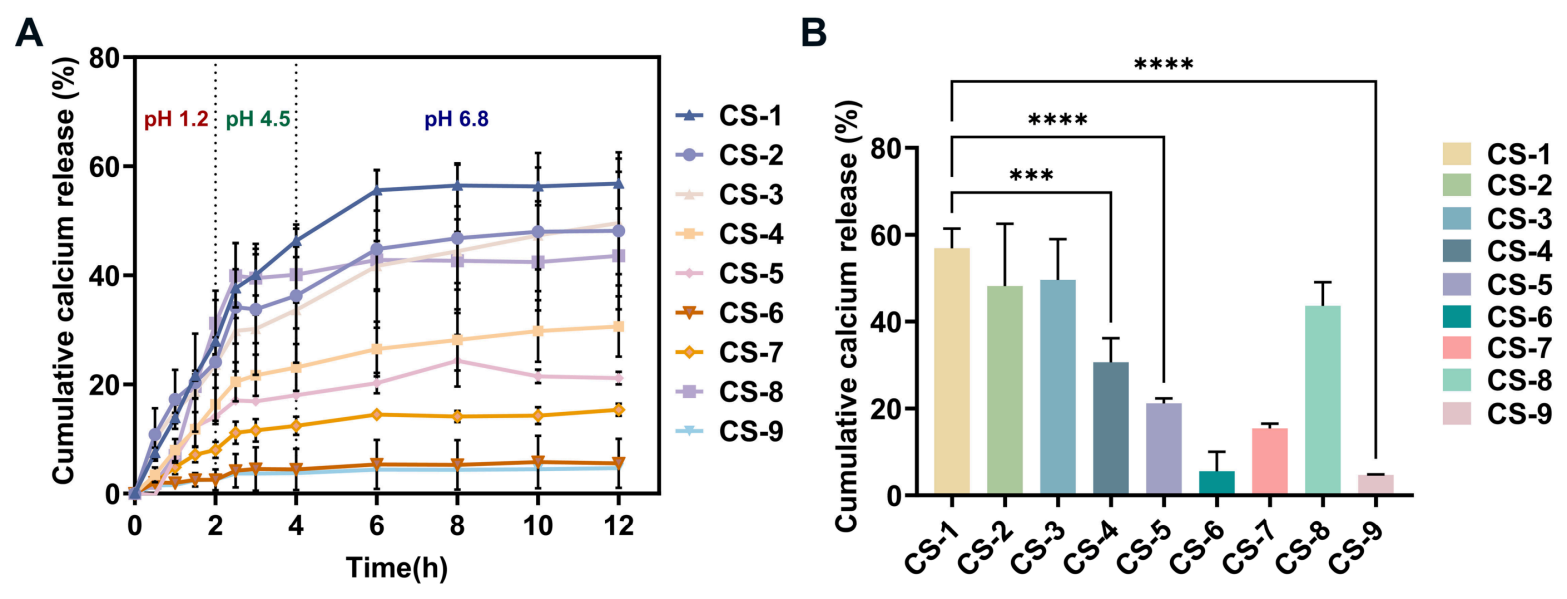
Cumulative calcium release profiles of different calcium formulations under sequential pH-gradient conditions simulating gastrointestinal transit. (**A**) Time-dependent cumulative calcium release profiles. (**B**) Cumulative calcium release for 12 h. Data are presented as mean ± SD (*n* = 3). *** *p* < 0.001, and **** *p* < 0.0001.

The dissolution profiles were further examined under sequential pH conditions designed to approximate gastrointestinal transit, with the medium changed from pH 1.2 to pH 4.5 and subsequently to pH 6.8. As shown in Figure 2A,B, most calcium formulations exhibited lower cumulative calcium release under the sequential pH condition than under the continuous pH 1.2 condition. This reduction became more apparent after the medium was shifted from the acidic phase to the higher-pH buffer systems, indicating that the increase in pH limited further calcium dissolution in several formulations. CS-1 maintained the highest cumulative calcium release throughout the experiment, and its release curve reached a plateau at a relatively high level after the pH transition. This suggests that calcium release from CS-1 was less affected by changes in medium pH. CS-2, CS-3, CS-4, and CS-9 showed intermediate release profiles, with cumulative release increasing gradually but remaining below that of CS-1. In contrast, CS-5, CS-6, CS-7, and CS-8 exhibited substantially lower release during the later phase of the experiment, particularly after the transition to pH 6.8. These results indicate that pH-dependent dissolution behavior contributed to the differences among calcium formulations, with CS-1 showing the highest cumulative calcium release under conditions approximating gastrointestinal transit.

### 2.2. In Vivo Pharmacokinetic Profiles of Calcium Formulations in Female Rats

#### 2.2.1. Plasma Calcium Concentration–Time Profiles After Oral Administration of Calcium Formulations

**Figure 3:**
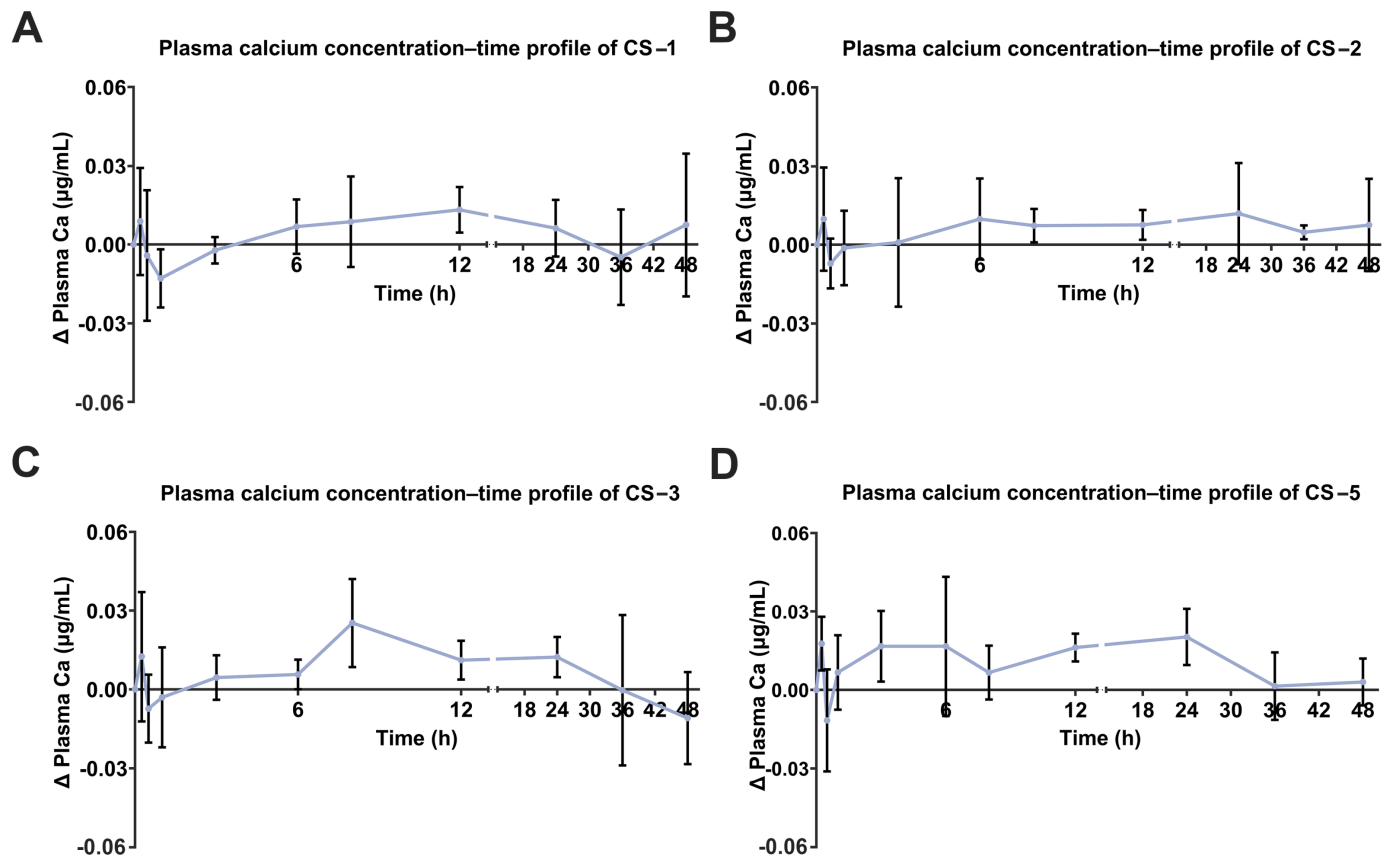
Plasma calcium concentration–time profiles of calcium formulations in female SD rats. (**A**) Plasma calcium concentration–time curve of CS-1. (**B**) Plasma calcium concentration–time curve of CS-2. (**C**) Plasma calcium concentration–time curve of CS-3. (**D**) Plasma calcium concentration–time curve of CS-5. Data are presented as mean ± SD (*n* = 3).

After oral administration of the calcium formulations at a calcium-equivalent dose of 82.5 mg/kg, baseline- and control-corrected plasma calcium concentration–time profiles were constructed over 48 h. As shown in Figure 3A–D, all four samples produced only moderate fluctuations in plasma calcium levels, which is consistent with the tight physiological regulation of systemic calcium homeostasis. Among the tested samples, CS-3 and CS-5 showed relatively higher increases in plasma calcium during the early-to-middle phase after administration, whereas CS-1 and CS-2 exhibited comparatively flatter plasma calcium profiles. However, the overall changes in plasma calcium remained limited across all groups, suggesting that plasma calcium concentration alone may not fully reflect differences in calcium absorption and utilization among the tested formulations.

#### 2.2.2. Apparent Calcium Absorption and Retention After Oral Administration of Calcium Formulations

**Figure 4:**
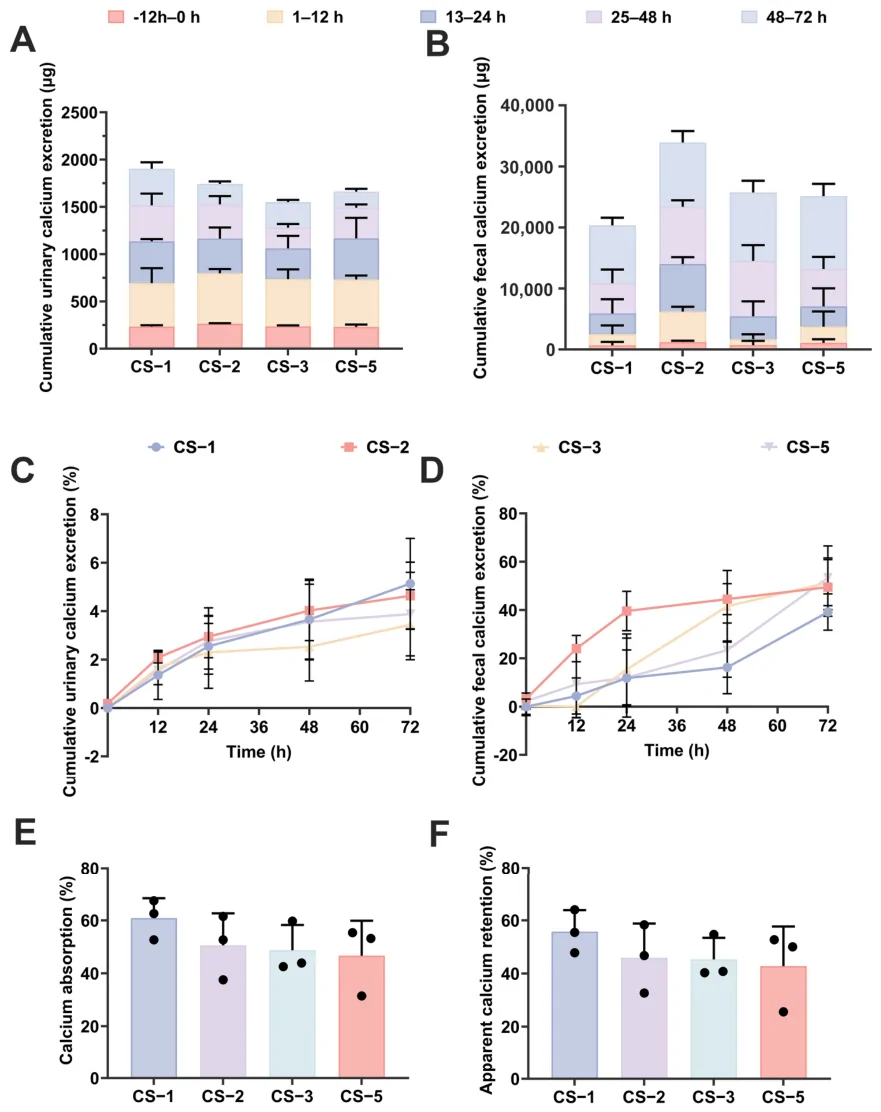
Calcium excretion, absorption, and apparent retention in female SD rats after intragastric administration of four calcium formulations at a dose of 82.5 mg/kg. (**A**) Cumulative urinary calcium excretion. (**B**) Cumulative fecal calcium excretion. (**C**) Cumulative urinary calcium excretion percentage. (**D**) Cumulative fecal calcium excretion percentage. (**E**) Calcium absorption. (**F**) Apparent calcium retention. The stacked bars in panels (**A**,**B**) represent calcium excretion during different collection intervals: −12–0 h, 1–12 h, 13–24 h, 25–48 h, and 48–72 h. Data are presented as mean ± SD (*n* = 3).

After intragastric administration of the four calcium formulations at a calcium-equivalent dose of 82.5 mg/kg, urinary and fecal calcium excretion were monitored over 72 h to evaluate calcium absorption and apparent retention. As shown in Figure 4A, urinary calcium excretion accumulated gradually across the different collection intervals in all treatment groups. Among the four formulations, CS-1 showed a relatively higher total urinary calcium excretion, whereas CS-3 exhibited a comparatively lowest urinary calcium output.

Fecal calcium excretion showed more evident differences among the formulations. As shown in Figure 4B, CS-2 exhibited the highest cumulative fecal calcium excretion, while CS-1 showed the lowest fecal calcium excretion over the 72 h period. Since fecal calcium mainly reflects the unabsorbed fraction of orally administered calcium, the lower fecal calcium loss observed in the CS-1 group suggests more efficient intestinal calcium absorption.

The cumulative excretion percentage profiles further supported these trends. As illustrated in Figure 4C, the cumulative urinary calcium excretion percentage increased gradually over time in all groups, with CS-1 reaching the highest value at 72 h. In contrast, the cumulative fecal calcium excretion percentage differed more clearly among groups (Figure 4D), with CS-1 maintaining a lower fecal excretion percentage than the other formulations during the later phase of the experiment.

Based on urinary and fecal calcium balance, calcium absorption and apparent calcium retention were further calculated. As shown in Figure 4E,F, CS-1 exhibited the highest calcium absorption and apparent calcium retention among the four tested formulations, whereas CS-3 and CS-5 showed relatively lower values. These results indicate that CS-1 had more favorable in vivo calcium utilization in female SD rats, characterized by reduced fecal calcium loss and improved apparent calcium absorption and retention.

### 2.3. In Vivo Pharmacodynamic Evaluation of Calcium Formulations in Retinoic Acid-Induced Osteoporotic Female ICR Mice

#### 2.3.1. Effects of Calcium Formulations on Femoral Trabecular Microarchitecture

**Figure 5:**
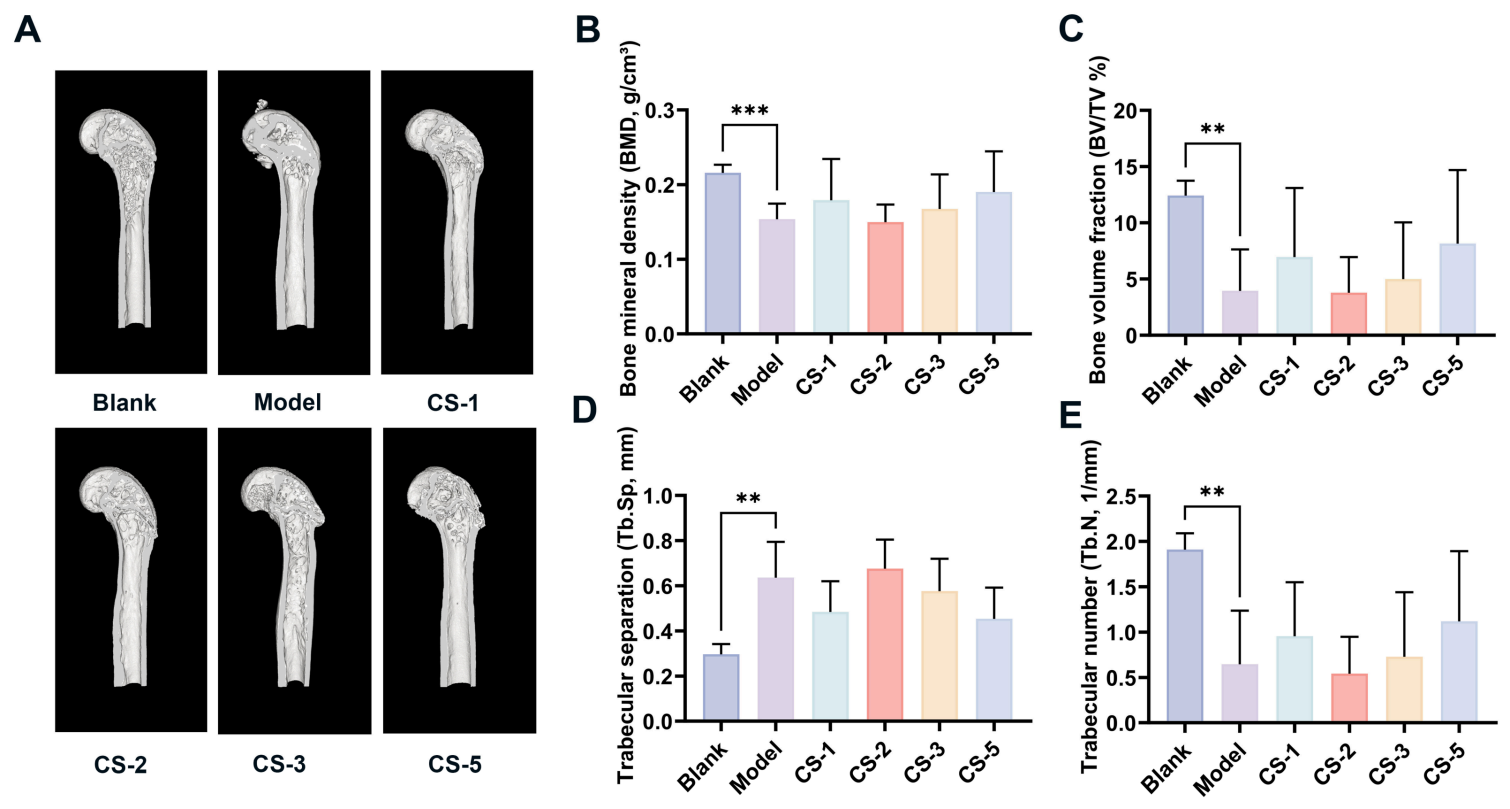
Effects of four calcium formulations on trabecular microarchitecture of femurs in osteoporotic ICR mice assessed by micro-computed tomography (micro-CT). (**A**) Representative micro-CT images of distal femur regions of interest (ROI) from mice treated with Blank (normal control), Model (osteoporotic control), and calcium formulations CS-1, CS-2, CS-3, and CS-5. (**B**–**E**) Quantitative micro-CT analysis of trabecular bone parameters: (**B**) bone mineral density (BMD, g/cm^3^), (**C**) bone volume fraction (BV/TV, %), (**D**) trabecular separation (Tb.Sp, mm), and (**E**) trabecular number (Tb.N, 1/mm). Data are presented as mean ± SD (*n* = 8). ** *p* < 0.01 and *** *p* < 0.001.

Micro-CT analysis was performed to evaluate the effects of different calcium formulations on femoral bone mineral density and trabecular microarchitecture in retinoic acid-induced osteoporotic ICR mice. As shown in Figure 5, the Model group exhibited marked deterioration of femoral trabecular microarchitecture compared with the Blank group. Quantitative analysis showed that BMD, BV/TV, and Tb.N were significantly decreased in the Model group, whereas Tb.Sp was significantly increased, indicating reduced bone mass, decreased trabecular abundance, and enlarged intertrabecular spaces after retinoic acid treatment. After calcium formulation intervention, the femoral microarchitectural parameters showed different response patterns among the treatment groups. For BMD, CS-1, CS-3, and CS-5 displayed numerically higher values than the Model group, with CS-5 showing the most apparent recovery tendency among the tested samples (Figure 5B). Similar trends were observed for BV/TV and Tb.N, where CS-1 and CS-5 showed partial increases compared with the Model group, although the values did not return to the level of the Blank group (Figure 5C,E). These results suggest that selected calcium formulations may partially preserve trabecular bone volume and trabecular number under osteoporotic conditions.

Trabecular separation was significantly increased in the Model group compared with the Blank group, suggesting disruption of trabecular spatial organization and enlargement of intertrabecular spaces (Figure 5D). Among the calcium formulation-treated groups, CS-5 showed the lowest mean Tb.Sp value, followed by CS-1, indicating a tendency toward improved trabecular spacing compared with the Model group. In contrast, CS-2 showed relatively limited recovery in the measured Micro-CT parameters. Representative three-dimensional micro-CT reconstructions further supported the quantitative findings (Figure 5A). The Blank group showed relatively dense and continuous trabecular architecture in the distal femur, whereas the Model group exhibited sparse and irregular trabeculae with enlarged intertrabecular spaces. After calcium formulation intervention, CS-1, CS-2, CS-3, and CS-5 showed partial restoration of proximal femoral trabecular architecture to varying degrees, with the visual changes generally consistent with the quantitative micro-CT parameters.

Overall, retinoic acid treatment induced marked impairment of femoral trabecular microarchitecture in ICR mice, characterized by decreased BMD, BV/TV, and Tb.N, together with increased Tb.Sp. Calcium formulation intervention produced variable effects on these parameters. Based on the descriptive trends, CS-5 showed the most consistent recovery tendency in trabecular microarchitectural indices, while CS-1 also showed partial improvement in several parameters.

#### 2.3.2. Effects of Calcium Formulations on Femoral Biomechanical Properties

**Figure 6:**
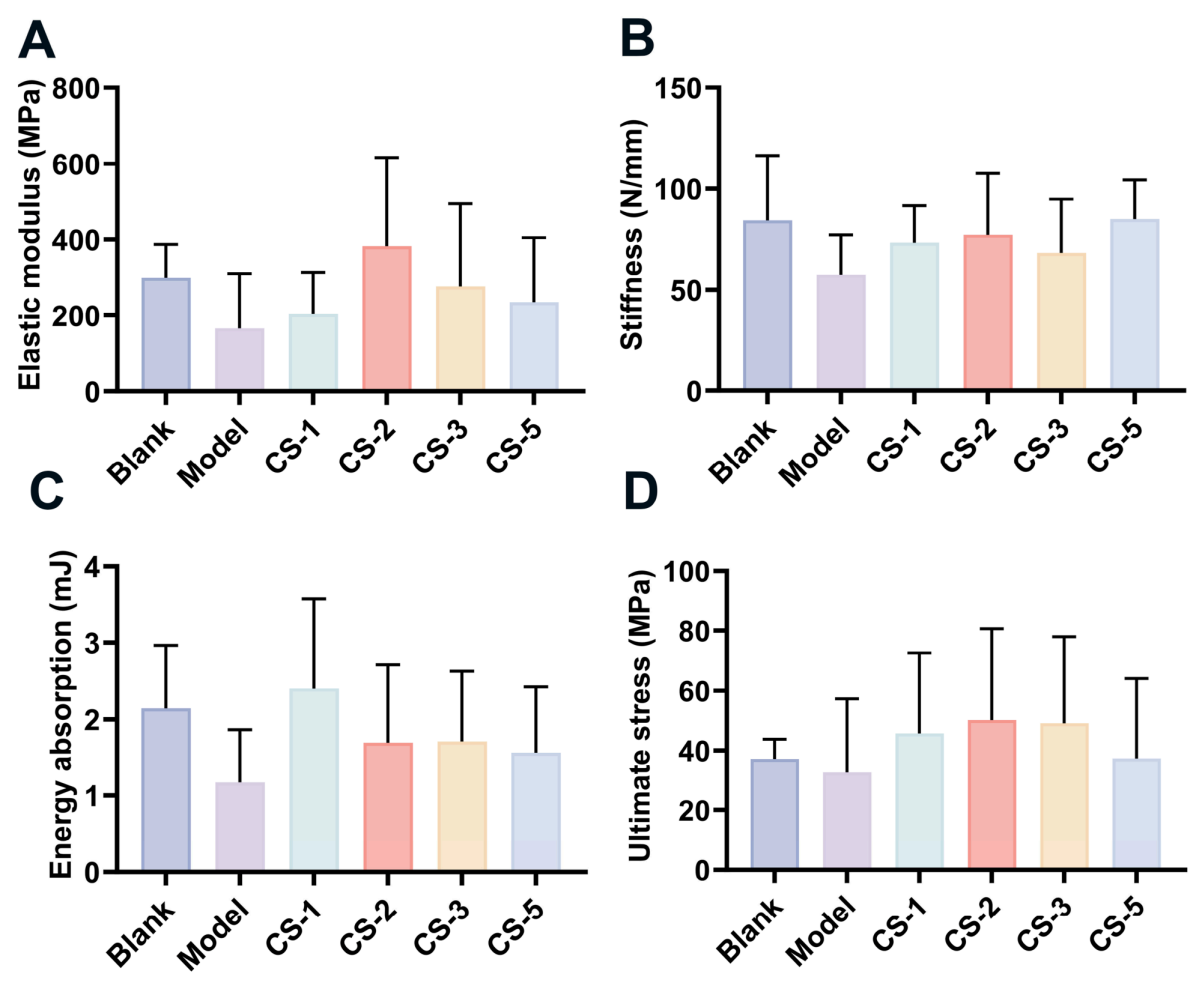
Three-point bending analysis of femoral biomechanical properties in retinoic acid-induced osteoporotic ICR mice treated with different calcium formulations. (**A**) Elastic modulus. (**B**) Stiffness. (**C**) Energy absorption. (**D**) Ultimate stress. Data are presented as mean ± SD. (*n* = 8).

Femoral biomechanical properties were evaluated using a three-point bending test to assess the effects of different calcium formulations on bone mechanical performance in retinoic acid-induced osteoporotic ICR mice. As shown in Figure 6, the Model group exhibited lower mean values of elastic modulus, stiffness, energy absorption, and ultimate stress than the Blank group, indicating impaired femoral mechanical properties following retinoic acid treatment. Following calcium formulation intervention, different biomechanical response patterns were observed among the treatment groups. For elastic modulus, CS-2 exhibited the highest mean value among all calcium formulation groups, followed by CS-5 and CS-3, whereas CS-1 showed a comparatively lower value (Figure 6A). For stiffness, all calcium formulation groups showed numerically higher values than the Model group, with CS-5 displaying the highest mean stiffness and approaching the level of the Blank group (Figure 6B). Energy absorption was reduced in the Model group compared with the Blank group. Among the calcium formulation groups, CS-1 showed the highest mean energy absorption value, whereas CS-3, CS-2, and CS-5 exhibited intermediate levels (Figure 6C). For ultimate stress, CS-2 and CS-3 demonstrated the highest mean values, followed by CS-1, while CS-5 showed a value similar to that of the Blank group (Figure 6D).

Overall, retinoic acid treatment was associated with deterioration of femoral biomechanical properties. Calcium formulation intervention partially improved these parameters, although the magnitude of improvement varied among formulations. Based on the descriptive data, CS-2 showed relatively favorable effects on elastic modulus and ultimate stress, CS-5 exhibited the highest stiffness, and CS-1 demonstrated the greatest energy absorption capacity. These findings suggest that different calcium formulations may differentially influence specific aspects of bone mechanical performance.

### 2.4. In Vivo Pharmacodynamic Evaluation of Calcium and Bone-Derived Calcium Formulations in Calcium-Deficient Female SD Rats

The preceding formulation-level evaluations showed that liquid calcium formulations exhibited favorable performance in terms of in vitro dissolution, in vivo calcium retention, and bone-protective efficacy. Among these formulations, CS-1 showed relatively superior overall performance. However, the pharmacodynamic advantage of CS-1 may not be attributable solely to its dosage form or calcium content, because salmon bone-derived calcium may also contain source-related mineral and peptide-associated components with potential osteogenic relevance. Therefore, in order to further clarify whether the biological effects observed are related to the source of its constituents, this study conducted an ingredient-level pharmacodynamic comparison in a calcium-deficient female SD rat model. Calcium citrate was used as a basic calcium-source control, while salmon bone calcium and several bone- or collagen-derived peptide ingredients were evaluated to compare their effects on skeletal growth, calcium homeostasis, trabecular microarchitecture, and femoral mechanical performance.

**Figure 7:**
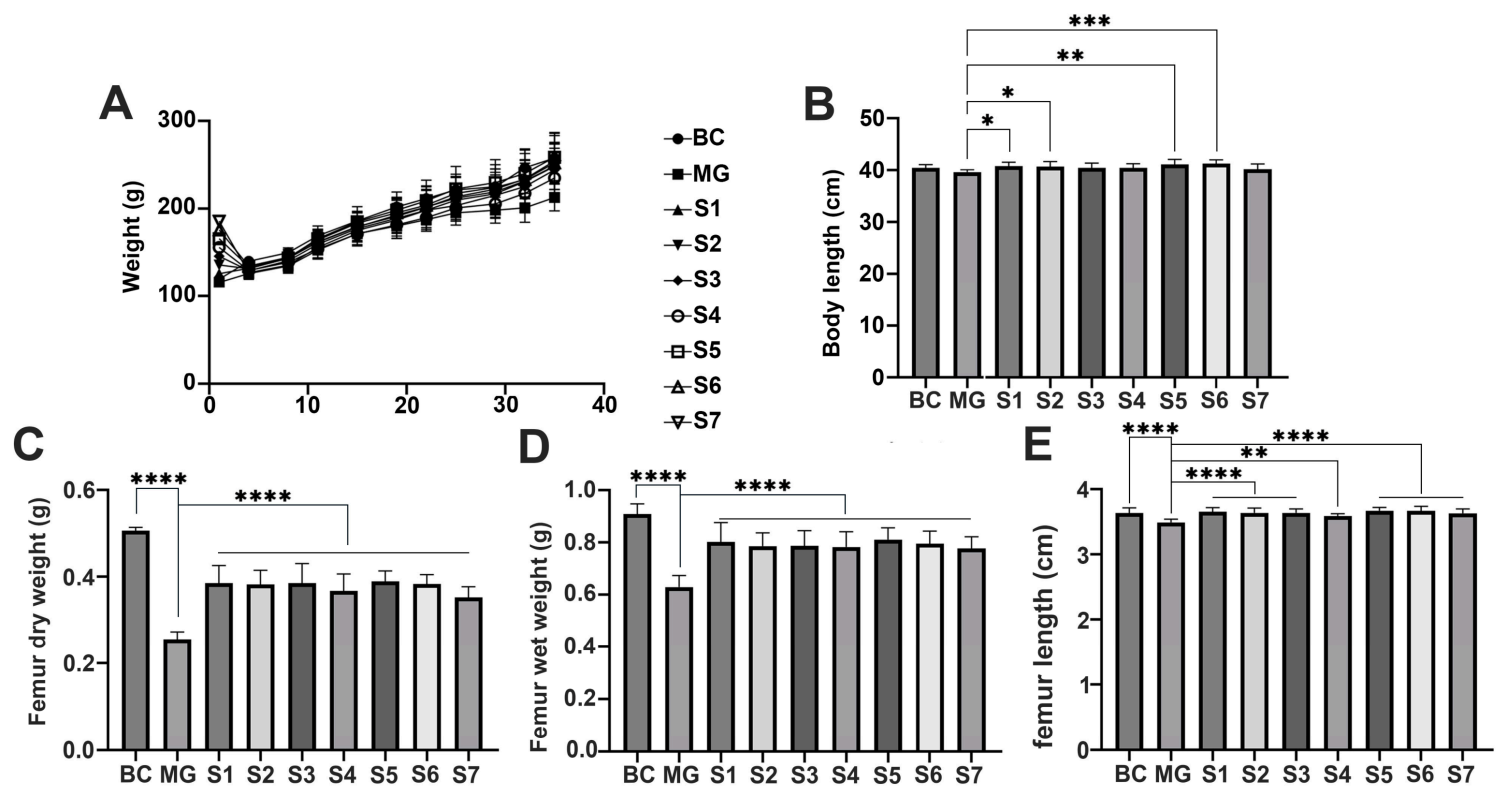
Effects of calcium and peptide-derived formulations on body weight, body length, and femoral growth-related parameters in calcium-deficient rats. (**A**) Body weight changes during the intervention period. (**B**) Body length at the experimental endpoint. (**C**) Femur dry weight. (**D**) Femur wet weight. (**E**) Femur length. BC (blank control); MG (model group); S1 (salmon bone calcium); S2 (collagen tripeptide); S3 (cod bone peptide); S4 (sturgeon cartilage peptide);S5 (bovine hide collagen peptide); S6 (bovine bone marrow collagen peptide); and S7 (calcium citrate). Data are presented as mean ± SEM. (*n* = 10). * *p* < 0.05, ** *p* < 0.01, *** *p* < 0.001, and **** *p* < 0.0001.

Body weight monitoring showed that all groups gained weight gradually during the intervention period, and no obvious abnormal growth pattern was observed among the treatment groups (Figure 7A). At the experimental endpoint, calcium deficiency impaired longitudinal growth and femoral development, as reflected by reduced body length, femur length, femur dry weight, and femur wet weight. Compared with the model group, calcium- and peptide-derived ingredient interventions increased these growth-related indices to varying degrees, suggesting partial alleviation of calcium-deficiency-associated skeletal growth retardation (Figure 7B–E).

**Figure 8:**
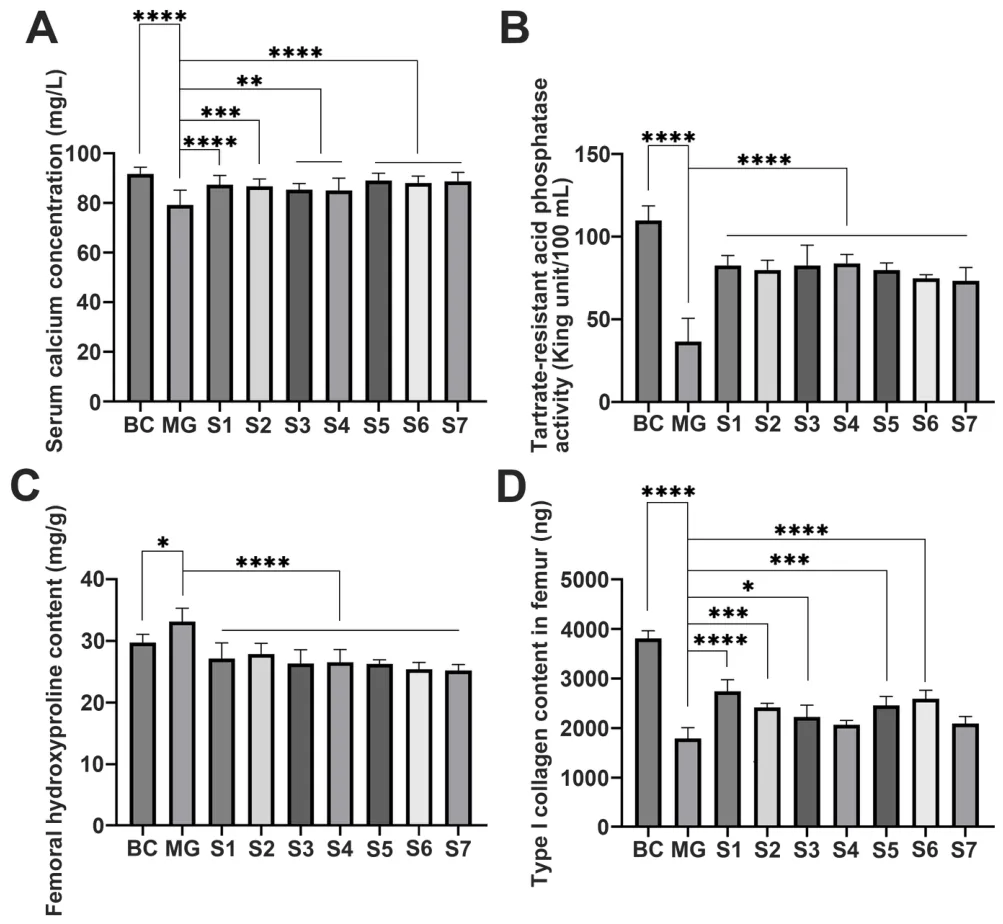
Effects of calcium and peptide-derived formulations on serum calcium, bone turnover-related enzyme activity, and femoral collagen metabolism in calcium-deficient rats. (**A**) Serum calcium concentration. (**B**) Tartrate-resistant acid phosphatase (TRAP) activity. (**C**) Femoral hydroxyproline content. (**D**) Type I collagen content in femur. BC (blank control); MG (model group); S1 (salmon bone calcium); S2 (collagen tripeptide); S3 (cod bone peptide); S4 (sturgeon cartilage peptide); S5 (bovine hide collagen peptide); S6 (bovine bone marrow collagen peptide); and S7 (calcium citrate). Data are presented as mean ± SEM. (*n* = 10). * *p* < 0.05, ** *p* < 0.01, *** *p* < 0.001, and **** *p* < 0.0001.

Serum and femoral biochemical analyses further indicated that calcium deficiency disturbed calcium homeostasis and bone matrix metabolism. The model group showed reduced serum calcium concentration, increased tartrate-resistant acid phosphatase activity, elevated femoral hydroxyproline content, and decreased femoral type I collagen content. These changes suggested enhanced bone resorption-related activity and impaired preservation of the collagen matrix under calcium-deficient conditions. After intervention, serum calcium levels increased, whereas TRAP activity and femoral hydroxyproline content decreased to different extents. Femoral type I collagen content was also partially restored, suggesting that calcium- and peptide-derived ingredients contributed to the maintenance of mineral homeostasis and collagen matrix integrity (Figure 8A–D).

**Figure 9:**
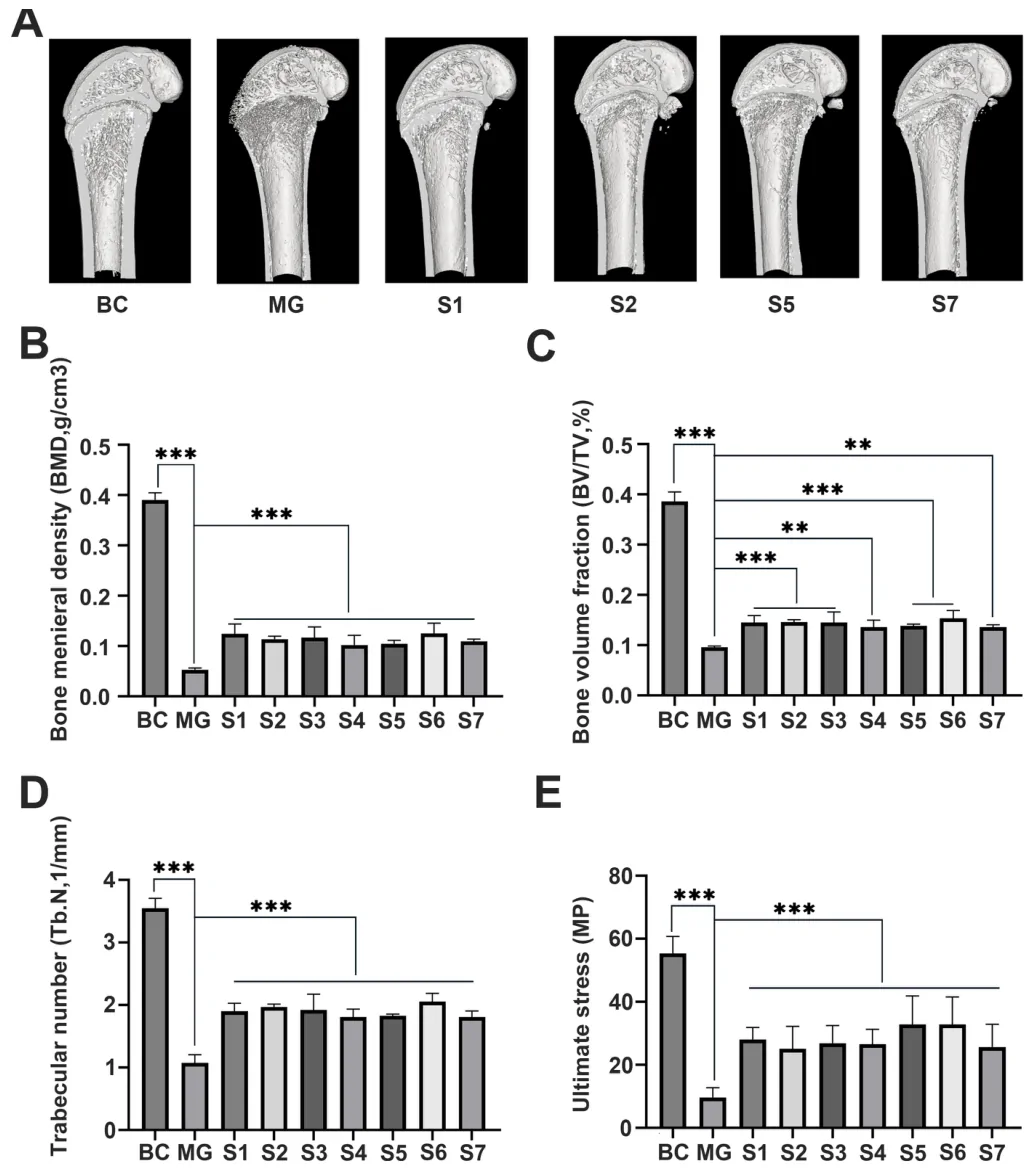
Effects of selected calcium and peptide-derived formulations on femoral microarchitecture and biomechanical properties in calcium-deficient rats. (**A**) Representative micro-CT images of distal femurs from the blank group, model group, salmon bone calcium group, collagen tripeptide group, bovine bone marrow collagen peptide group, and calcium citrate group. (**B**) Bone mineral density (BMD). (**C**) Bone volume fraction (BV/TV). (**D**) Trabecular number (Tb.N). (**E**) Ultimate stress BC (blank control); MG (model group); S1 (salmon bone calcium); S2 (collagen tripeptide); S3 (cod bone peptide); S4 (sturgeon cartilage peptide); S5 (bovine hide collagen peptide); S6 (bovine bone marrow collagen peptide); and S7 (calcium citrate). Data are presented as mean ± SEM. (*n* = 10). ** *p* < 0.01, *** *p* < 0.001.

Representative micro-CT images showed marked deterioration of proximal femoral trabecular architecture in the model group compared with the blank group, characterized by reduced trabecular abundance and a more porous trabecular network (Figure 9A). In contrast, salmon bone calcium, collagen tripeptide, bovine bone marrow collagen peptide, and calcium citrate partially preserved the trabecular structure. Quantitative micro-CT analysis was consistent with these morphological observations. Compared with the model group, the intervention groups showed significant improvements in BMD, BV/TV, and Tb.N to varying degrees, indicating partial restoration of femoral trabecular bone mass and microarchitecture (Figure 9B–D). Importantly, although multiple calcium- or peptide-derived ingredients produced statistically significant improvements relative to the model group, the magnitude of recovery differed among sources, suggesting that the bone-protective effect was source-dependent rather than a uniform response to calcium supplementation alone. Femoral biomechanical testing further showed that calcium deficiency reduced bone mechanical competence, as reflected by the decrease in ultimate strength in the model group. Treatment with calcium- and peptide-derived ingredients improved ultimate strength to varying degrees, indicating that the restoration of trabecular microarchitecture was accompanied by partial recovery of femoral resistance to mechanical loading (Figure 9E). Among the representative ingredients shown in the main figure, salmon bone calcium and bovine bone marrow collagen peptide exhibited relatively stronger recovery trends in both micro-CT parameters and biomechanical performance, whereas calcium citrate, as a basic calcium-source control, produced moderate improvement. These results suggest that bone-derived calcium sources and peptide-associated components may provide additional skeletal benefits beyond simple calcium replenishment.

Overall, the ingredient-level comparison demonstrated that different calcium sources and bone-derived peptide components exerted distinguishable pharmacodynamic effects in calcium-deficient rats. The significant improvements observed across several treatment groups indicate that calcium supplementation and peptide-derived components can both contribute to skeletal recovery. More importantly, the differences in recovery magnitude among groups support a source-dependent efficacy profile. Together with the preceding formulation-level findings, these results provide pharmacodynamic evidence that thesuperior overall performance of CS-1 may be associated not only with its liquid formulation characteristics but also with the biological contribution of salmon bone-derived calcium components. This ingredient-level comparison therefore provides a rational basis for the comprehensive efficacy ranking of different calcium sources and supports the selection of salmon bone calcium-containing formulations for further development.

## 3. Discussion

The evaluation of calcium formulations is often based on labeled calcium content, in vitro dissolution, or a single absorption parameter. However, calcium release, maintenance of calcium solubility in the gastrointestinal tract, intestinal absorption, and subsequent bone mineralization represent distinct stages of calcium utilization [26]. Therefore, no single parameter adequately reflects the overall performance of a calcium formulation. In the present study, commercially available calcium formulations were systematically compared in terms of in vitro calcium release, in vivo calcium utilization, and bone-protective efficacy. The aim was not to rank individual products but to examine how dosage form, calcium source, and functional ingredients contribute to differences in formulation performance. Unlike conventional comparisons focused primarily on calcium content or dissolution, the present study links formulation-dependent calcium release with in vivo calcium utilization and bone-protective efficacy in animal models within a single evaluation framework. Its scientific contribution therefore lies not in identifying a superior commercial product, but in demonstrating that formulation performance should be interpreted across the sequential stages of calcium release, absorption and retention, and bone-protective effects, while considering the combined influences of dosage form, calcium source, and functional ingredients.

Distinct calcium release profiles were observed under simulated gastric and gastrointestinal pH conditions. These differences were associated, at least in part, with dosage form. Liquid formulations contain calcium in a dissolved or highly dispersed state and therefore bypass tablet disintegration, matrix erosion, or capsule shell dissolution, resulting in more rapid calcium release [27,28]. In contrast, calcium release from solid formulations depends on multiple formulation characteristics, including disintegration, excipient composition, particle size, and calcium salt solubility [29]. However, dosage form alone did not account for the observed differences. Although CS-5 was a tablet, it exhibited one of the highest calcium release rates among the solid formulations, indicating that formulation design and calcium salt selection are also important determinants of release behavior. Previous studies have similarly reported that calcium citrate is less dependent on gastric acidity than calcium carbonate and generally exhibits more favorable dissolution characteristics under conditions of reduced gastric acid secretion [30,31,32].

Despite differences in calcium release, plasma calcium concentrations showed only minor changes after administration. In contrast, fecal calcium, urinary calcium, apparent calcium absorption, and apparent calcium retention more effectively differentiated calcium utilization among formulations. This finding is consistent with the tight physiological regulation of systemic calcium homeostasis. Plasma calcium is maintained within a narrow range through coordinated regulation of intestinal absorption, renal excretion, skeletal buffering, parathyroid hormone, and vitamin D [33]. Consequently, short-term differences in calcium absorption are not necessarily reflected by circulating calcium concentrations. Apparent calcium absorption and retention therefore provide more sensitive indicators of calcium utilization than plasma calcium alone [34]. This may explain why improved calcium release did not produce marked elevations in plasma calcium but was associated with reduced fecal calcium loss and increased calcium retention.

Among the tested formulations, CS-1 consistently showed favorable calcium release and apparent calcium utilization. Because several liquid calcium formulations were included in the study, these findings cannot be attributed solely to the liquid dosage form. Compared with the other liquid formulations, CS-1 additionally contains salmon bone-derived ingredients, which may contribute to its performance [25,35]. Bone-derived minerals and collagen-derived peptides have been reported to interact with Ca^2+^ through carboxyl, amino, hydroxyl, or phosphorylated functional groups [36,37], thereby stabilizing soluble calcium and reducing precipitation with phosphate, oxalate, or fatty acids [38,39]. Similar mechanisms have been described for casein phosphopeptides, collagen peptides, calcium-binding peptides [40], and peptide–calcium chelates, all of which improve calcium solubility and intestinal availability. Taken together, the superior performance of CS-1 is more plausibly explained by the combined effects of liquid delivery, calcium salt characteristics, and salmon bone-derived functional components than by dosage form alone.

The reductions in body length, femur length, and femoral wet and dry weights in the model group further indicate that calcium deficiency impaired skeletal growth and bone mass accumulation. Previous studies in growing rats have similarly associated low calcium intake with smaller skeletal dimensions and altered physicochemical properties of the femur [41,42]. The partial recovery of these indices after calcium- and peptide-based interventions therefore suggests an attenuation of calcium-deficiency-related skeletal growth retardation. Nevertheless, because these gross indices are also influenced by overall somatic growth, they should be interpreted together with the micro-CT and biomechanical results. The biochemical findings provide additional evidence of altered bone turnover. Increased TRAP activity is consistent with enhanced osteoclast-related resorptive activity, although the osteoclast specificity of this marker is greatest for the TRAP5b isoform [43]. Type I collagen constitutes the principal organic framework of bone and contributes substantially to its mechanical integrity; thus, its reduction indicates impaired preservation of the bone matrix [44]. Hydroxyproline is closely associated with collagen and is commonly used to assess collagen content or turnover, but it cannot independently distinguish bone formation from bone resorption. Therefore, the reductions in TRAP activity and femoral hydroxyproline, together with the partial restoration of type I collagen after treatment, are more appropriately interpreted as partial normalization of bone resorption and collagen-matrix turnover. This interpretation is consistent with a recent study in low-calcium-fed female mice showing that peptide–calcium complexes increased bone type I collagen and Col1a1 expression while improving bone mass and microarchitecture [45].

The results obtained with CS-5 further support this interpretation. Although formulated as a tablet, CS-5 exhibited relatively efficient calcium release and was therefore selected for the in vivo study as a representative solid formulation with relatively favorable calcium release. This finding indicates that appropriately designed solid formulations can achieve satisfactory calcium release despite the inherent limitations of conventional dosage forms. Consequently, the comparison between CS-1 and CS-5 primarily reflects differences between a liquid delivery system and an optimized solid formulation, whereas the comparison among liquid formulations highlights the potential contribution of salmon bone-derived ingredients.

The pharmacodynamic results further demonstrate that in vitro calcium release alone cannot predict bone-protective efficacy. Across both animal models, calcium supplementation partially improved bone mineral content, trabecular microarchitecture, and mechanical properties. These outcomes are influenced not only by calcium availability but also by bone remodeling, osteoblast and osteoclast activity, collagen matrix metabolism, and skeletal adaptation [46,47,48]. Thus, efficient calcium release appears to be necessary but is not sufficient to determine skeletal efficacy.

Differences in pharmacodynamic responses among calcium citrate and bone-derived peptide formulations further suggest that peptide components may contribute to bone protection beyond their effects on calcium release. Bone-derived collagen peptides may enhance calcium bioavailability by maintaining calcium in a soluble form while simultaneously providing amino acids involved in extracellular matrix synthesis and bone remodeling. Previous studies have shown that fish bone collagen peptide–calcium chelates promote calcium transport and osteogenic differentiation, whereas bovine bone collagen peptide–calcium complexes exhibit calcium-binding activity and stimulate osteoblast function [49,50]. Consistent with these reports, the salmon bone calcium formulation and bovine bone marrow collagen peptide formulation showed similar improvements in trabecular microarchitecture and femoral mechanical strength, implying that bone-derived peptides may contribute to skeletal protection through mechanisms beyond calcium supplementation alone.

Nevertheless, the present study does not establish a direct molecular interaction between salmon bone-derived components and calcium citrate. The available data support the possibility that calcium citrate provides a readily soluble calcium source, whereas salmon bone-derived peptides help maintain calcium in a soluble state, reduce precipitation, or facilitate intestinal calcium transport. However, these mechanisms remain speculative and require further investigation.

In summary, this study suggests that the performance of calcium formulations is jointly influenced by dosage form, calcium source, excipient composition, and functional ingredients [51,52]. The advantages of CS-1 may arise from the combined effects of the liquid dosage form and salmon bone-derived functional components, rather than from the effect of the delivery system alone; the favorable release performance of CS-5 indicates that well-optimized solid dosage forms can still serve as effective comparators. A limitation of this study is that commercially available products and bone-derived combination formulations inherently contain multiple variables, making it impossible to completely separate the independent contributions of dosage form, calcium source, and functional peptides. A further limitation is that the CS-5 tablet was ground and suspended in 1% sodium carboxymethylcellulose before oral gavage. Although this approach maintained the original formulation composition and calcium dose, it inevitably disrupted the physical structure of the tablet and may have altered its disintegration and dissolution behavior compared with intact tablet administration. In addition, the apparent calcium absorption and retention rates were calculated based on fecal and urinary calcium excretion and therefore remain indirect estimates. Future studies should employ composition-controlled model formulations and incorporate analyses of peptide–calcium complexation, intestinal transport, and molecular markers of bone metabolism to further verify the specific relationships among liquid delivery systems, salmon bone-derived components, and calcium salt forms. These findings indicate that dosage-form design, calcium-source selection, and bone-derived functional ingredients may all affect the in vivo utilization and bone-protective effects of calcium formulations. Therefore, an integrated evaluation strategy encompassing in vitro release, in vivo retention, and bone-related effects may be adopted in the future development and evaluation of calcium formulations [40]. The findings also suggest that fish bone-derived liquid calcium formulations and calcium–peptide composite formulations warrant further investigation for the development and formulation optimization of bone health products [53].

## 4. Materials and Methods

### 4.1. Materials and Reagents

This study selected nine commercially available samples for in vitro dissolution testing and labeled them CS-1 through CS-9 according to a predetermined analytical sequence. All samples had clearly labeled ingredients and dosage forms. To examine the influence of dosage form, four liquid formulations (CS-1–CS-4), three tablets (CS-5–CS-7), and two soft capsules (CS-8 and CS-9) were included. To explore the influence of calcium source, representative products containing calcium citrate, calcium carbonate, calcium hydroxyapatite, and salmon bone-derived calcium were selected. Formulation composition was also considered, including representative products containing vitamin D and K/K_2_, casein phosphopeptides, undenatured type II collagen, magnesium, and zinc. These samples were selected to cover different formulation characteristics rather than to represent all calcium products available on the market.

In the subsequent in vivo evaluation, CS-1, CS-2, CS-3, and CS-5 were selected as representative samples after comprehensively considering dosage form representativeness, formulation differences, in vitro dissolution performance, and sample quantity control in animal experiments. CS-1 through CS-3 were all liquid calcium formulations with different ingredients that reflect the in vivo performance of different liquid calcium formulations; CS-5 is a tablet sample with good in vitro calcium release performance and was therefore selected as the representative reference for traditional solid calcium formulations. The dosage form, sample category, and experimental use of each coded sample are summarized in Table 1. To avoid disclosure of product-specific commercial information, all tested commercial samples are referred to only by their assigned codes throughout the manuscript.

Hydrochloric acid (analytical grade; Nanjing Chemical Reagent Co., Ltd., Nanjing, China), anhydrous sodium acetate (analytical grade; Nanjing Chemical Reagent Co., Ltd., Nanjing, China), potassium dihydrogen phosphate (analytical grade; Shanghai Lingfeng Chemical Reagent Co., Ltd., Shanghai, China), sodium hydroxide (analytical grade; Xilong Scientific Co., Ltd., Shantou, China), glacial acetic acid (analytical grade, approximately 17.4 mol/L; Nanjing Chemical Reagent Co., Ltd., Nanjing, China), calcium carbonate reference standard (99.95–100%; Tianjin Chemical Reagent Research Institute, Tianjin, China), lanthanum chloride heptahydrate (LaCl_3_·7H_2_O, analytical grade; Sinopharm Chemical Reagent Co., Ltd., Shanghai, China), sodium carboxymethylcellulose (grade: SJJ-L; pharmaceutical-grade excipient, Anhui Shanhe Pharmaceutical Excipients Co., Ltd., Huainan, China), and deionized water were used for the preparation of dissolution media and calcium determination. Electronic grade G2 nitric acid (CAS No. 7697-37-2) was used for sample digestion. A low-calcium diet (code: 21392.95) was purchased from Jiangsu Synergetic Pharmaceutical Bioengineering Co., Ltd. (Nanjing, China). Retinoic acid (CAS No. 302-79-4) was purchased from Shanghai Aladdin Biochemical Technology Co., Ltd. (Shanghai, China) and used to establish the experimental osteoporosis model. Unless otherwise specified, all reagents were of analytical grade.

### 4.2. Animals and Ethical Approval

Female SD rats weighing 250–300 g were used for the in vivo pharmacokinetic evaluation of the selected calcium formulations. The rats were obtained from the Nanjing Qinglongshan Animal Breeding Base (Nanjing, China). After a three-day quarantine and acclimatization period, the animals were fed a low-calcium diet for one week before administration to reduce the influence of dietary calcium intake on the assessment of calcium exposure, excretion, and retention.

Female ICR mice weighing 25–30 g were used for the pharmacodynamic evaluation of the four representative calcium formulations, namely CS-1, CS-2, CS-3, and CS-5. The mice were obtained from the Nanjing Qinglongshan Animal Breeding Base (Nanjing, China). Animals were housed under standard laboratory conditions with free access to food and water and were acclimatized for one week before the experiment. The anti-osteoporotic efficacy of the selected calcium formulations was evaluated in a retinoic-acid-induced osteoporotic mouse model.

The animal use protocol was approved by China Pharmaceutical University Ethics Committee and in compliance with guidelines of the National Institute of Health Guide for the Care and Use of Laboratory Animals (approval no. 2023-06-004) The study animals were housed in a pathogen-free facility with a 12 h light/dark cycle and given access to food and water ad libitum.

### 4.3. In Vitro Dissolution Study of Calcium Formulations

The in vitro dissolution behavior of the nine calcium formulations, coded as CS-1 to CS-9, was evaluated using a SOTAX CE7 flow-through cell dissolution system (SOTAX AG, Aesch, Switzerland) [54,55]. The study was designed to characterize calcium release under acidic gastric conditions and under sequential pH conditions approximating gastrointestinal transit. All dissolution experiments were performed using a 0.8 μm filter membrane, a medium volume of 900 mL, and a constant flow rate of 4 mL/min.

Two dissolution protocols were employed. In the gastric-condition protocol, samples were exposed to pH 1.2 hydrochloric acid solution for 12 h. In the sequential gastrointestinal pH protocol, dissolution was initiated in pH 1.2 hydrochloric acid solution for the first 2 h, followed by replacement with pH 4.5 acetate buffer for the next 4 h and subsequently with pH 6.8 phosphate buffer until the end of the 12 h experiment. This pH-transition design was used to assess whether differences in dosage form and formulation composition influenced calcium release during exposure to changing gastrointestinal environments [54,55,56].

Dissolution samples were automatically collected at 0.5, 1, 1.5, 2, 2.5, 3, 4, 6, 8, 10, and 12 h, with 1 mL withdrawn at each time point. The cumulative release calculation was corrected for the calcium removed during previous sampling. Prior to calcium quantification, the collected samples were diluted with deionized water in the presence of 8.0% lanthanum chloride solution to minimize matrix interference during atomic absorption analysis. Soft capsule and liquid calcium formulations were diluted 25-fold, whereas tablet samples were diluted 50-fold to ensure that the calcium concentration remained within the linear range of the calibration curve.

Calcium concentrations in the dissolution samples were determined by an AA-7000 atomic absorption spectrophotometry (Shimadzu Corporation, Kyoto, Japan) using a calcium hollow cathode lamp at 422.7 nm [57,58]. Calibration standards were prepared over a concentration range of 2.0–30.0 μg/mL using calcium standard solution and the same lanthanum chloride matrix. Calcium concentrations were calculated from the calibration curve, and the dissolution profiles were expressed as the cumulative percentage of calcium released relative to the declared calcium content of each sample.

### 4.4. Pharmacokinetic Study of Different Calcium Formulations

The in vivo calcium exposure and excretion profiles of selected calcium formulations were evaluated in female SD rats [59]. Based on the coding sequence used for the calcium formulation products, four representative samples, namely CS-1, CS-2, CS-3, and CS-5, were included in the pharmacokinetic assessment. After a three-day quarantine and acclimatization period, the rats were maintained on a low-calcium diet for one week to reduce dietary calcium interference before administration [59].

Before administration, endogenous calcium levels were monitored by collecting plasma, fecal, and urinary samples at 24 h intervals, and calcium concentrations were confirmed to remain stable using an Agilent 5800 inductively coupled plasma optical emission spectrometer (ICP-OES; Agilent Technologies Trading (Shanghai) Co., Ltd., Shanghai, China) at a Ca emission wavelength of 396.847 nm. [59,60]. The rats were then randomly assigned to the calcium formulation treatment groups and a control group. Prior to administration of CS-5, prepare a 1% aqueous solution of carboxymethylcellulose sodium (CMC-Na) as a suspending agent [61]. Grind the CS-5 tablets into a fine powder, then weigh out the appropriate amount of tablet powder based on the equivalent elemental calcium dose. Disperse the powder in the 1% CMC-Na solution and vortex thoroughly to prepare a uniform suspension; this suspension should be prepared immediately before use. Liquid samples should be diluted to the same equivalent elemental calcium dose before administration. Calcium formulations were administered by oral gavage at a calcium dose of 82.5 mg/kg. Animals were fasted for 6 h before dosing, with free access to water throughout the experiment.

To characterize systemic calcium exposure after administration, whole blood samples were collected into heparinized tubes before dosing and at 15 min, 30 min, 1 h, 3 h, 6 h, 8 h, 12 h, 24 h, 36 h, and 48 h after dosing. Plasma was obtained by centrifugation at 8000 rpm for 5 min and stored at −20 °C until analysis. To evaluate calcium excretion, feces and urine were collected during the pre-dose period and over the post-dose intervals of −12–0 h and the post-dose intervals of 1–12, 13–24, 25–48, and 48–72 h. The collected samples were stored at −80 °C before ICP-OES analysis [60].

Calcium concentrations in plasma, feces, and urine were quantified by ICP-OES after appropriate sample pretreatment. Plasma samples were digested with nitric acid and diluted before analysis. Fecal samples were dried to constant weight at 70 °C, ground, sieved, accurately weighed, and subjected to nitric acid digestion prior to dilution and measurement. Urine samples were centrifuged, and the supernatants were diluted with 5% nitric acid solution before analysis. Plasma calcium concentration–time profiles were constructed for each treatment group, while fecal and urinary calcium excretion were calculated over each collection interval. To minimize the influence of endogenous calcium homeostasis, plasma calcium concentrations were corrected using both the pre-dose baseline and the control group values before pharmacokinetic comparison [62].

### 4.5. Pharmacodynamic Study of Different Calcium Formulations

#### 4.5.1. Establishment of the Retinoic Acid-Induced Osteoporotic Model and Calcium Formulation Intervention

After acclimatization, osteoporosis-like bone deterioration was induced by repeated oral administration of retinoic acid. Except for the normal control group, mice were administered 0.9% retinoic acid suspension by gavage at a dose of 90 mg/kg once daily for 21 consecutive days. Mice in the normal control group received an equal volume of physiological saline during the same period.

After model establishment, the retinoic acid-treated mice were randomly assigned into five groups, including the model control group and four calcium formulation intervention groups receiving CS-1, CS-2, CS-3, or CS-5. The pretreatment method for the tablets is the same as described in Section 4.4, “Pharmacokinetic Study of Different Calcium Formulations.” The calcium formulations were administered by gavage once daily for 21 days at a calcium-equivalent dose of 65 mg/kg. The normal control and model control groups received an equal volume of vehicle. This experimental design was used to compare the bone-protective effects of different calcium formulations under the same osteoporotic condition.

#### 4.5.2. Femur Collection and Sample Preparation

At the end of the intervention period, mice were euthanized according to the experimental schedule, and bilateral femurs were collected for ex vivo analysis. During sample collection, the surrounding muscles, fascia, and residual connective tissues were carefully removed, while preserving the integrity of the periosteum and bone structure. The isolated femurs from each animal were allocated for micro-computed tomography analysis and three-point bending mechanical testing.

Femurs assigned for micro-CT analysis were fixed in paraformaldehyde at 4 °C for 48 h and then stored at 4 °C until scanning. Before scanning, residual fixative on the sample surface was gently removed, and no additional pretreatment was performed. Femurs used for biomechanical testing were wrapped in saline-moistened gauze immediately after soft tissue removal, sealed to prevent dehydration, and stored at −80 °C until analysis. Before testing, frozen samples were thawed naturally to room temperature while being kept moist [63]. Repeated freeze–thaw cycles and prolonged ex vivo exposure were avoided to minimize potential effects on bone microstructure and mechanical properties [63].

#### 4.5.3. Micro-CT Analysis of Femoral Microarchitecture

Femoral microarchitecture was evaluated using a micro-computed tomography (CT) scanner (Bruker SkyScan 1276, Billerica, MA, USA). Image reconstruction was performed using NRecon software (version 1.7.4), and quantitative analysis was conducted using CTAn software (version 1.20.3). The scanning parameters for mouse femurs were set at 55 kV and 200 μA, with a spatial resolution of 6 μm.

The distal femoral trabecular region was selected as the region of interest. After three-dimensional reconstruction, images were segmented using a consistent threshold across all groups. Bone mineral density (BMD) and trabecular microstructural parameters, including bone volume fraction (BV/TV), trabecular thickness (Tb.Th), and trabecular separation (Tb.Sp), were calculated to characterize the effects of the calcium formulation interventions on cancellous bone architecture.

#### 4.5.4. Femoral Three-Point Bending Test in Retinoic Acid-Induced Osteoporotic ICR Mice

The biomechanical properties of the femur were assessed using a three-point bending test performed with a 5940 series single-column material testing system. After thawing to room temperature, each femur was placed horizontally on two supports with a span length of 4 mm. A vertical load was applied to the mid-diaphyseal region at a crosshead speed of 0.5 mm/min until a visible fracture occurred or the recorded load rapidly decreased to near zero [64].

Load–displacement curves were recorded using the instrument software. Elastic modulus, stiffness, energy absorption, and maximum stress were calculated to evaluate the effects of different calcium formulation interventions on femoral mechanical performance.

### 4.6. Pharmacodynamic Study of Different Bone Peptides

#### 4.6.1. Establishment of the Calcium-Deficient Bone Loss Model and Intervention Design

The pharmacodynamic effects of different bone-derived peptide preparations were evaluated using a calcium-deficient diet-induced bone loss model in growing female SD rats [65]. Specific pathogen-free female SD rats, four weeks of age, were obtained from SPF Biotechnology Co., Ltd. (Suzhou, China; license No. SCXK (Su) 2022-0006). After one week of acclimatization, 90 rats were randomly assigned to nine groups, with ten animals in each group.

Rats in the normal control group were fed a standard calcium diet, whereas all other groups were maintained on a calcium-deficient diet throughout the intervention period. The diets were prepared according to the AIN-93 purified rodent diet formulation, with the standard calcium diet containing 0.6% calcium and 0.65% phosphorus, and the calcium-deficient diet containing 0.0% calcium and 0.65% phosphorus [66]. The calcium-deficient diet was stored at −20 °C and equilibrated to room temperature before use.

The experimental groups included a normal control group, a calcium-deficient model group, a calcium citrate group, and six intervention groups receiving calcium citrate in combination with different bone- or collagen-derived peptide formulations. The normal control and model groups received 0.5% sodium carboxymethyl cellulose solution by gavage. The calcium citrate group received calcium citrate alone at 595.1 mg/kg. The salmon bone calcium group received calcium citrate and salmon bone calcium at 465.0 and 133.3 mg/kg, respectively. The remaining peptide intervention groups received calcium citrate at 595.1 mg/kg in combination with collagen tripeptide, cod bone peptide, sturgeon cartilage peptide, bovine hide collagen peptide, or bovine bone marrow collagen peptide at 133.3 mg/kg [67]. All interventions were administered once daily by gavage for five weeks.

#### 4.6.2. Physiological Monitoring and Biological Sample Collection

During the intervention period, general physiological conditions, including fur appearance, tooth condition, activity, and mental status, were monitored regularly [68,69]. Body weight was recorded twice weekly, and food intake was recorded three times per week. Body length was measured before and after the intervention to assess growth-related changes under calcium-deficient conditions.

At the end of the five-week intervention, rats were anesthetized and blood samples were collected from the abdominal aorta. Whole blood samples were allowed to stand for 30 min and then centrifuged at 3500 rpm for 10 min to obtain serum. Serum samples were stored at −80 °C until biochemical analysis [70,71]. After blood collection, bilateral femurs were isolated, and adherent muscles and ligament tissues were carefully removed. Femoral wet weight, bone length, and gross appearance were recorded before further allocation for microstructural, biomechanical, mineral, and bone matrix analyses.

For each group, four left femurs were randomly selected for micro-CT analysis and subsequently used for histological staining after fixation. The remaining six left femurs were used for three-point bending mechanical testing. Six right femurs were used for dry weight, calcium, phosphorus, and hydroxyproline determination, whereas the remaining four right femurs were used for type I collagen analysis [72,73,74,75,76].

#### 4.6.3. Biochemical Evaluation of Serum and Bone Composition

Serum alkaline phosphatase and tartrate-resistant acid phosphatase activities were determined using commercial assay kits according to the manufacturers’ instructions. Serum calcium and phosphorus concentrations were quantified by ICP-OES [71].

For bone mineral analysis, right femurs were dried at 105 °C until constant weight, cooled to room temperature, and weighed to obtain bone dry weight. Dried femoral samples were digested with nitric acid, followed by acid removal at 130 °C, dilution with deionized water, and elemental analysis by ICP-OES to determine bone calcium and phosphorus contents.

Bone matrix-related indices were further assessed by measuring type I collagen and hydroxyproline contents in femoral tissue. For type I collagen analysis, femoral samples were pulverized under liquid nitrogen, and bone protein was extracted using a commercial bone tissue protein extraction kit. The supernatant was collected and analyzed by enzyme-linked immunosorbent assay. For hydroxyproline determination, dried femoral samples were ground into powder, digested, and analyzed by liquid chromatography.

#### 4.6.4. Micro-CT and Histological Analysis of Femoral Bone

Femoral microarchitecture was evaluated by ex vivo micro-computed tomography [72,77]. Fixed femoral specimens were scanned, and the proximal femoral region was selected for quantitative analysis after calibration of CT values. Trabecular bone parameters, including bone mineral density, bone volume, total bone volume, bone volume fraction, trabecular number, trabecular thickness, and trabecular separation, were calculated to assess the effects of different peptide interventions on cancellous bone architecture [72,73,77].

After micro-CT scanning, fixed femoral specimens were rinsed with phosphate-buffered saline and decalcified in 4.13% EDTA-2Na solution at pH 7.4 and 4 °C for 30 days. After complete decalcification, samples were washed under running water, embedded in paraffin, sectioned along the median sagittal plane of the distal femur, and stained with hematoxylin and eosin and Masson’s trichrome staining. Histological sections were examined under a microscope to assess bone tissue morphology and collagen matrix organization [78].

#### 4.6.5. Femoral Three-Point Bending Test in Calcium-Deficient SD Rats

The biomechanical properties of femurs were evaluated using a three-point bending test. Before testing, frozen left femurs were thawed to room temperature and rehydrated with physiological saline to minimize dehydration-related changes in mechanical performance. The external long and short diameters at the mid-diaphysis were measured three times and averaged before mechanical loading.

Each femur was placed on a material testing system equipped with a DBSL-3t sensor, with a support span of 8 mm. Load was applied at the mid-diaphysis at a loading speed of 0.01 mm/s until fracture. After fracture, the internal long and short radii at the fracture site were measured three times and averaged. Load–deflection data were converted to stress–strain curves, from which bone material parameters related to stiffness and toughness were calculated to characterize femoral mechanical performance after different peptide interventions [73,74].

### 4.7. Statistical Analysis

All quantitative data are presented as the mean ± standard deviation (SD) or mean ± standard error (SEM), unless otherwise stated. Statistical analyses were performed using GraphPad Prism 10.1.2 software. The normality of data distribution and homogeneity of variance were assessed before group comparisons. For comparisons among multiple groups, one-way analysis of variance (ANOVA) followed by Tukey’s multiple comparison test was used when the data satisfied parametric assumptions. When the assumptions of normality or equal variance were not met, a non-parametric Kruskal–Wallis test followed by Dunn’s multiple comparison test was applied.

## 5. Conclusions

This study evaluated the in vitro dissolution behavior, in vivo calcium utilization, and pharmacodynamic effects of different calcium formulations and calcium/bone-derived peptide formulations. The tested samples showed distinct calcium release profiles under simulated gastric and sequential gastrointestinal pH conditions, indicating that calcium release was influenced by both formulation characteristics and medium pH. Among the calcium formulations, CS-1 showed relatively rapid and sustained calcium release and exhibited higher apparent calcium absorption and retention in rats, suggesting more favorable calcium utilization under the present experimental conditions.

In retinoic acid-induced osteoporotic ICR mice, calcium formulation intervention partially improved femoral trabecular microarchitecture and biomechanical properties, although the effects differed among samples and across evaluation indices. In the calcium-deficient female SD rat model, calcium and bone-derived peptide formulations improved several bone-related parameters, including femoral growth characteristics, serum calcium homeostasis, bone turnover markers, femoral mineral deposition, bone matrix-related indices, and trabecular microarchitecture. These findings suggest that the tested formulations may contribute to bone protection by improving calcium availability and supporting both mineral deposition and bone matrix maintenance.

Overall, the study results indicate that a single endpoint cannot comprehensively evaluate the efficacy of calcium formulations. In vitro dissolution, calcium absorption and retention, serum biochemical parameters, bone mineral composition, micro-CT parameters and biomechanical properties should be considered comprehensively to assess the overall performance of calcium-based formulations. The current preliminary findings provide experimental evidence supporting the potential use of liquid calcium formulations and osteogenic peptide formulations to enhance calcium utilization and mitigate calcium-deficiency-related bone degeneration; however, the respective contributions of the dosage form and functional components require further investigation.

## Figures and Tables

**Table 1:** Characteristics and declared compositions of the commercial calcium formulations evaluated in this study.

Sample Code	Dosage Form	Calcium Source	Blended Ingredients
CS-1	Liquid	Calcium citrate, calcium from salmon bones	Vitamin D_3_, vitamin K_2_, undenatured type II collagen
CS-2	Liquid	Calcium citrate	Vitamin D, freeze-dried apple powder, dl-α -tocopherol
CS-3	Liquid	Calcium citrate	Casein phosphopeptides, vitamin D, vitamin K, vitamin C, zinc
CS-4	Liquid	Calcium citrate	Magnesium, zinc, vitamin D_3_, vita-min K_2_
CS-5	Tablet	Calcium citrate	Vitamin D_3_, vitamin K_2_
CS-6	Tablet	Calcium hydrogen phosphate	Vitamin D_3_, glucosamine hydrochloride, dimethyl sulphone (MSM), chondroitin sulphate sodium
CS-7	Tablet	Calcium carbonate	Vitamin D_3_, vitamin K_2_
CS-8	Soft capsule	Calcium carbonate	Vitamin K_2_, vitamin D_3_
CS-9	Soft capsule	Calcium hydroxyphosphate	Vitamin D_3_, vitamin K_2_

## Data Availability

The original contributions presented in this study are included in the article/Supplementary Material. Further inquiries can be directed to the corresponding authors.

## Supplementary Materials

The following supporting information can be downloaded at: https://www.mdpi.com/article/10.3390/ph19081172/s1, Figure S1. Effects of calcium and peptide-derived formulations on body weight, body length, and femoral growth-related parameters in calcium-deficient rats. (A) Body weight changes during the intervention period. (B) Body length at the experimental endpoint. (C) Femur dry weight. (D) Femur wet weight. (E) Femur length. Data are presented as mean ± SD. Statistical significance was analyzed by appropriate multiple comparisons test. * *p* < 0.05, ** *p* < 0.01, *** *p* < 0.001, and **** *p* < 0.0001; Figure S2. Effects of calcium and peptide-derived formulations on serum calcium, bone turnover-related enzyme activity, and femoral collagen metabolism in calcium-deficient rats. (A) Serum calcium concentration. (B) Tartrate-resistant acid phosphatase (TRAP) activity. (C) Femoral hydroxyproline content. (D) Type I collagen content in femur. Data are presented as mean ± SD. Statistical significance was analyzed by appropriate multiple comparisons test. * *p* < 0.05, ** *p* < 0.01, *** *p* < 0.001, and **** *p* < 0.0001.
