# Supplementary material for: Analysis of Dissolution, Pharmacokinetic, and Bone-Protective Differences Among Calcium Formulations with Different Dosage Forms and Calcium Sources
Source: Pharmaceuticals (Basel). 2026 Jul 27;19(8):1172. doi: 10.3390/ph19081172 (PMC13516845; doi:10.3390/ph19081172)
Supplement: Supplementary file 1 [file pharmaceuticals-19-01172-s001.zip › pharmaceuticals-4399183-supplementary.pdf]

Supporting Information

Analysis of Dissolution, Pharmacokinetic, and Bone-Protective Differences Among Calcium Formulations with Different Dosage Forms and Calcium Sources

Mengxi Wang [1], Shuo Liu [1], Guang Wei [1], Haiyang Wang [1], Zewei Huang [2], Yang Ding [1,*] and Guochen Han [1,*]

[1] State Key Laboratory of Natural Medicines, Department of Pharmaceutics, China Pharmaceutical University, Nanjing 210009, China; wmx202108@163.com (M.W.); ls2608732869@163.com (S.L.); 15951073609@126.com (G.W.); 3325010507@stu.cpu.edu.cn (H.W.)

[2] Sichuan Institute of Food Inspection, Chengdu 610097, China; huangzewei@scsspjyyjy.wecom.work

* Correspondence: dydszyzf@163.com (Y.D.); guochenhan24@163.com (G.H.)

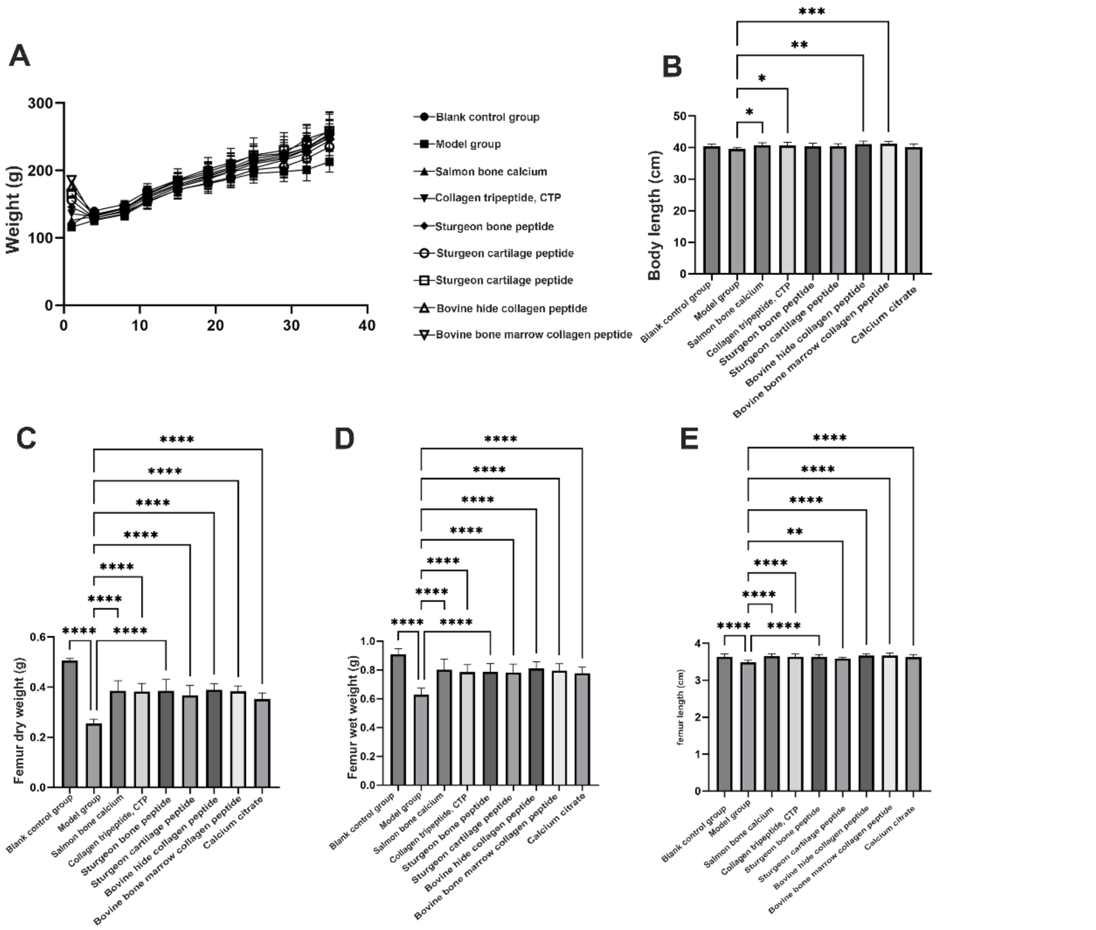

Supplementary Figure S1. Effects of calcium and peptide-derived formulations on body weight, body length, and femoral growth-related parameters in calcium-deficient rats. (A) Body weight changes during the intervention period. (B) Body length at the experimental endpoint. (C) Femur dry weight. (D) Femur wet weight. (E) Femur length. Data are presented as mean ± SD. Statistical significance was analyzed by appropriate multiple comparisons test. *$p < 0.05$, **$p < 0.01$, ***$p < 0.001$, and ****$p < 0.0001$

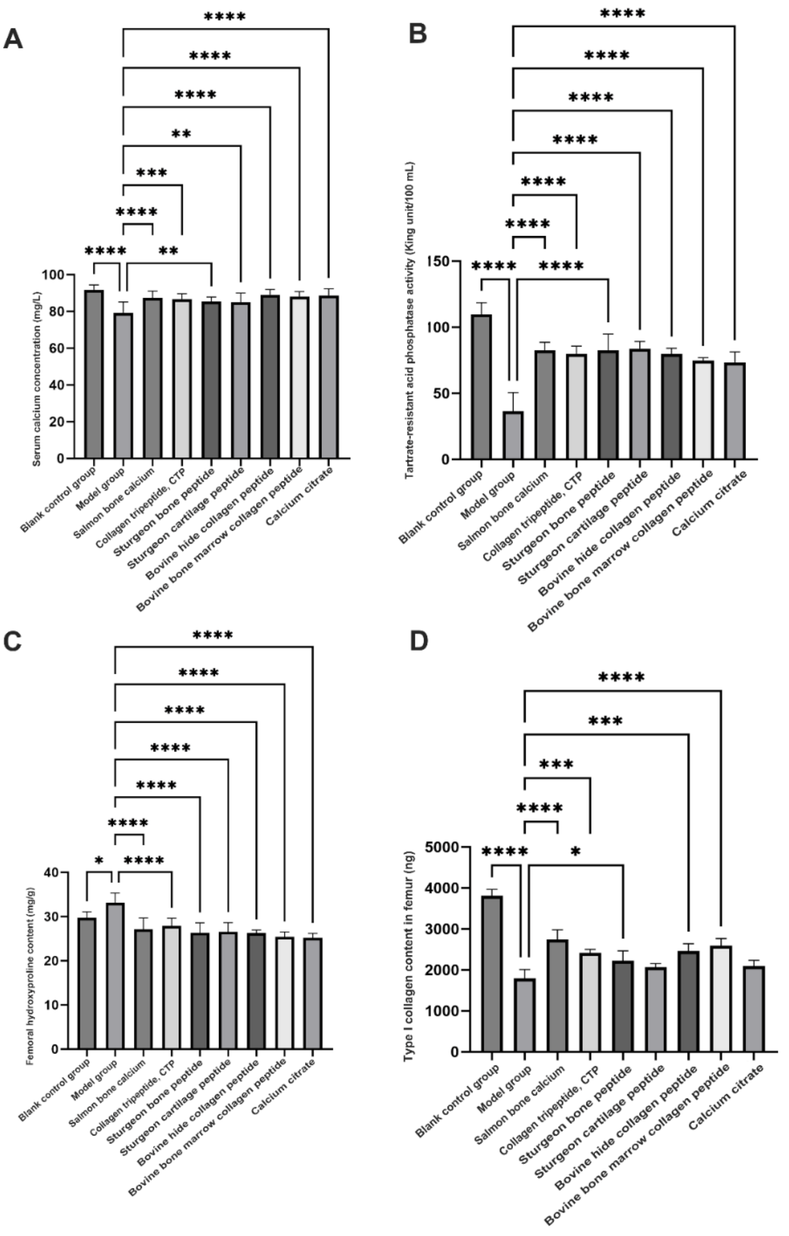

Supplementary Figure S2. Effects of calcium and peptide-derived formulations on serum calcium, bone turnover-related enzyme activity, and femoral collagen metabolism in calcium-deficient rats. (A) Serum calcium concentration. (B) Tartrate-resistant acid phosphatase (TRAP) activity. (C) Femoral hydroxyproline content. (D) Type I collagen content in femur. Data are presented as mean ± SD. Statistical significance was analyzed by appropriate multiple comparisons test. *$p < 0.05$, **$p < 0.01$, ***$p < 0.001$, and ****$p < 0.0001$.
